# Plant species composition and local habitat conditions as primary determinants of terrestrial arthropod assemblages

**DOI:** 10.1007/s00442-023-05345-6

**Published:** 2023-03-03

**Authors:** Cynthia Tobisch, Sandra Rojas-Botero, Johannes Uhler, Jörg Müller, Johannes Kollmann, Christoph Moning, Martin Brändle, Martin M. Gossner, Sarah Redlich, Jie Zhang, Ingolf Steffan-Dewenter, Caryl Benjamin, Jana Englmeier, Ute Fricke, Cristina Ganuza, Maria Haensel, Rebekka Riebl, Lars Uphus, Jörg Ewald

**Affiliations:** 1 Institute of Ecology and Landscape, Weihenstephan-Triesdorf University of Applied Sciences, Freising, Germany; 2 Chair of Restoration Ecology, School of Life Sciences, Technical University of Munich, Freising, Germany; 3 Field Station Fabrikschleichach, Department of Animal Ecology and Tropical Biology, Julius-Maximilians-University Würzburg, Würzburg, Germany; 4 Bavarian Forest National Park, Grafenau, Germany; 5 Division of Animal Ecology, Department of Ecology, Philipps-Universität Marburg, Marburg, Germany; 6 Forest Entomology, Swiss Federal Institute for Forest, Snow, and Landscape Research WSL, Birmensdorf, Switzerland; 7 Department of Environmental Systems Science, Institute of Terrestrial Ecosystems, ETH Zürich, Zürich, Switzerland; 8 Department of Animal Ecology and Tropical Biology, Julius-Maximilians-University Würzburg, Würzburg, Germany; 9 Ecoclimatology, School of Life Sciences, Technical University of Munich, Freising, Germany; 10 Professorship of Ecological Services, Bayreuth Center of Ecology and Environmental Research (BayCEER), University of Bayreuth, Bayreuth, Germany

**Keywords:** Community ecology, Ellenberg indicator values, Functional groups, Plant–insect interactions, Species-environment relationships

## Abstract

Arthropods respond to vegetation in multiple ways since plants provide habitat and food resources and indicate local abiotic conditions. However, the relative importance of these factors for arthropod assemblages is less well understood. We aimed to disentangle the effects of plant species composition and environmental drivers on arthropod taxonomic composition and to assess which aspects of vegetation contribute to the relationships between plant and arthropod assemblages. In a multi-scale field study in Southern Germany, we sampled vascular plants and terrestrial arthropods in typical habitats of temperate landscapes. We compared independent and shared effects of vegetation and abiotic predictors on arthropod composition distinguishing between four large orders (Lepidoptera, Coleoptera, Hymenoptera, Diptera), and five functional groups (herbivores, pollinators, predators, parasitoids, detritivores). Across all investigated groups, plant species composition explained the major fraction of variation in arthropod composition, while land-cover composition was another important predictor. Moreover, the local habitat conditions depicted by the indicator values of the plant communities were more important for arthropod composition than trophic relationships between certain plant and arthropod species. Among trophic groups, predators showed the strongest response to plant species composition, while responses of herbivores and pollinators were stronger than those of parasitoids and detritivores. Our results highlight the relevance of plant community composition for terrestrial arthropod assemblages across multiple taxa and trophic levels and emphasize the value of plants as a proxy for characterizing habitat conditions that are hardly accessible to direct environmental measurements.

**Supplementary Information:**

The online version contains supplementary material available at 10.1007/s00442-023-05345-6.

## Introduction

There is strong evidence of ongoing arthropod losses worldwide (van Klink et al. 2020; Wagner 2020), with local declines in terrestrial arthropod diversity reported in many regions of Central Europe (Habel et al. 2016; Seibold et al. 2019; Barendregt et al. 2022). Local arthropod diversity and community composition are negatively affected by land-use intensification (Allan et al. 2014; Deguines et al. 2016; Gossner et al. 2016). This involves losses of habitat and floral resources, in particular for specialized insects (Scheper et al. 2014; Abrahamczyk et al. 2020). Diversity patterns of plants and arthropods are clearly linked (Zhang et al. 2016; Ebeling et al. 2018) and declines in arthropod species richness are associated with reduced plant species richness (Haddad et al. 2009). However, the mechanisms through which characteristics of vegetation influence arthropod assemblages are less well understood. To derive effective conservation measures benefitting arthropods, it is crucial to better understand the key drivers of arthropod diversity and community composition.

Numerous studies examined the relationships between plant and arthropod communities, but most of them used species richness or other univariate diversity measures that do not describe the actual species compositions of the studied groups (Schaffers et al. 2008), or include measures of species composition only for plants, but not arthropods (e.g., Schuldt et al. 2019). Those studies considering the influence of plant species composition on arthropod assemblage composition often consider only one or few arthropod groups (Sanderson et al. 1995; Müller et al. 2011; Zellweger et al. 2017), or do not account for other environmental factors potentially involved (Zhang et al. 2016).

Schaffers et al. (2008) conducted the first study assessing effects of multiple environmental factors, including vegetation structure and species composition, soil, climate and surrounding landscape, on the assemblages of seven taxonomic arthropod groups in seminatural grassland in the Netherlands. They found that local plant species’ composition best explained species composition of all investigated arthropod taxa. Since the studied groups mainly consisted of first- and second-order consumers, the influence of plant species composition on the assemblage composition of other trophic groups such as parasitoids or detritivores remains to be tested. Moreover, it is unclear how much direct trophic interactions between arthropods and food plants contribute to the high explanatory power of plant species composition for arthropod assemblages.

Arthropods strongly respond to climatic conditions such as temperature, since they are ectothermic organisms (Chown and Nicolson 2004; Angilletta 2009). Several studies emphasized climate as a key driver of arthropod communities, reporting significant changes in species composition along gradients of temperature and precipitation (Lessard et al. 2011; Uhey et al. 2020). In addition, responses of arthropods to climate can indirectly be driven by temperature ranges of other organisms such as host plants (Hodkinson 2005), or microclimatic effects mediated by vegetation structure (Suggitt et al. 2011; Prather and Kaspari 2019). Studies considering both independent and combined effects of climate and vegetation on arthropod communities are lacking (but see Zellweger et al. 2017).

Arthropods depend on habitat structures that provide food, shelter or nesting sites. As habitat requirements vary strongly among taxa, the presence and type of habitat structures is an important filter for arthropod species composition (Lengyel et al. 2016). Land use determines the type of vegetation providing such habitat structures. Therefore, changes in arthropod community composition are often due to changes in land cover (e.g., Birkhofer et al. 2015; Deguines et al. 2016; Seibold et al. 2019). Blake et al. (2003) classified data on ground beetles from more than 400 sites in Scotland into 14 different assemblages. Each of these was clearly linked to one distinct primary vegetation type, such as mire, woodland, or arable land. Since these vegetation types very much correspond to land-cover classes used in remote sensing, the question arises of how well general habitat information predicts arthropod assemblages compared to detailed information on plant species composition.

Besides determining the habitat structure, plants form the basis of arthropod food webs, providing resources for numerous species feeding on foliage, nectar, and pollen. Many phytophagous insects directly depend on a few or even a single plant species or genus (Bernays and Graham 1988; Forister et al. 2015), and approximately one-third of the bees in Central Europe are oligolectic or monolectic (Bogusch et al. 2020). Plant species composition should thus be a strong predictor of herbivore and pollinator assemblages since it determines the availability of food plants.

Vegetation has a strong integrative character in the sense that plant community composition reflects local abiotic conditions such as temperature, soil moisture, light, or nutrient availability (Diekmann 2003). Ellenberg indicator values are a widely used tool to extract this environmental information from floristic data across Europe (Ellenberg et al. 1991; Diekmann 2003; Bartelheimer and Poschlod 2016). Responses of arthropod assemblages to vegetation composition may partly be responses to abiotic conditions reflected by plants but are difficult to measure directly (Schaffers et al. 2008; Müller et al. 2011; Zellweger et al. 2017). To what extent these integrative effects of environmental conditions mirrored in vegetation composition are relevant to arthropod assemblages is unknown.

In the present study, we sampled vascular plants and terrestrial arthropods across large-scale gradients of climate and land use, covering four different habitat types typical for landscapes in Central Europe, i.e., forest, grassland, arable field, and settlement (Redlich et al. 2021). Using Malaise traps for arthropod sampling, we obtained an extensive dataset that enabled us to consider a broad range of arthropod taxa and trophic levels. DNA metabarcoding allowed taxonomic determination on the same level of identification for all sampled organisms (Uhler et al. 2021). The study aimed to disentangle the effects of plant species composition and environmental drivers on arthropod composition and to assess which aspects of vegetation contribute to the relationships between plant and arthropod assemblages. Specifically, we asked the following:

1. Is plant species composition the most important determinant of arthropod assemblage composition (as opposed to land cover and climate)?
2. Does the influence of plant species composition decrease with increasing trophic levels?
3. Is the relationship between plant and arthropod assemblages more driven by direct dependencies, as relationships between monophagous arthropods and food plants, or indirect dependencies, as local abiotic conditions reflected by vegetation?

## Materials and methods

**Fig. 1:**
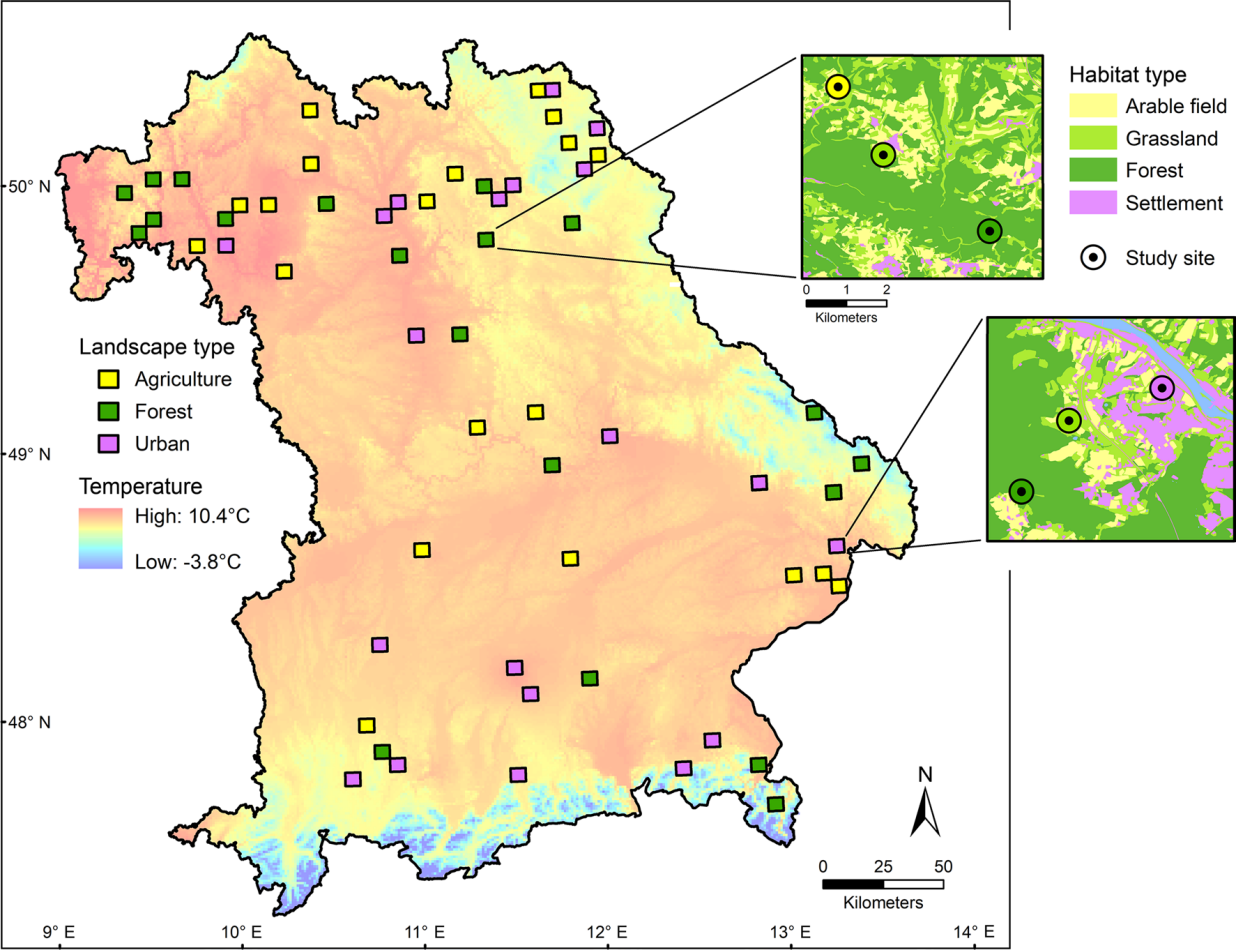
Distribution of 60 landscapes (5.8 km × 5.8 km) within the federal state Bavaria. Insets show examples of a forest- and an urban-dominated landscape containing three study sites representing different local habitat types

We selected 60 landscapes corresponding to topographical map quadrants (5.8 km × 5.8 km grid cell size) throughout the federal state of Bavaria, Southern Germany. Following a stratified design, the selected landscapes covered five climate zones which were defined based on the mean annual temperature for the period 1981–2010 (< 7.5, 7.5–8.0, 8.1–8.5, 8.5–9, > 9 °C) as well as three different landscape-scale land-use types (urban, agricultural, and forest-dominated landscapes; Fig. 1). Nested within each landscape, we established three study sites of 3 m × 30 m in the most dominant habitat types (out of four possible habitat types: forest, grassland, arable field, and settlement; Fig. 1), with finally 179 study sites (one of initially 180 study sites was not established due to denial of landowner permission). Site locations were standardized to open patches dominated by herbaceous vegetation within or – in case of arable fields – next to the respective habitat type. Forest sites were thus established within forest glades or clearings. The sites were spatially distributed across an area of approximately 300 km × 300 km and covered an elevational range of 162–1122 m above sea level. For detailed information on study design and site selection, see Redlich et al. (2021).

### Arthropod sampling and metabarcoding

Arthropods were captured using Malaise traps at the center of all 179 sites. Traps were emptied every 2 weeks from mid-April 2019 until mid-August 2019. DNA metabarcoding based on CO1-5P (mitochondrial cytochrome oxidase 1) was conducted for a subset of samples from three periods of major insect activity (second half of May, June, and July, respectively), following the bioinformatic methods described in Hausmann et al. (2020). Based on the Barcode of Life Data System (Ratnasingham and Hebert 2007), we used BINs (Barcode Index Numbers) as taxonomic units, since this classification shows close concordance with species (Ratnasingham and Hebert 2013) and allows a comparable level of species identification for all orders. Overall, 97% of all BINs identified in the Malaise traps were included in the analysis (7301 out of 7496). These BINs represented 361 arthropod families from 26 different taxonomic orders. While flying insects represented the major fraction of BINs identified in our traps, the samples also contained non-flying arthropods such as spiders (97 BINs in total) and carabid beetles (74 BINs). According to their taxonomic classification, BINs were assigned to five functional groups (herbivores, pollinators, predators, parasitoids including parasites, and detritivores) based on literature (Table S1). Families with unknown information, with many genera of different functional groups, or omnivorous families were excluded from functional group classification. Taxa that are phytophages as larvae and feed on pollen and nectar as adults (as is the case for Lepidoptera) were assigned to both herbivores and pollinators. The relative proportions of trophic groups in our dataset (49% herbivores, 12% predators, 18% parasitoids, 14% detritivores) approximately corresponded to the functional composition of the entire German insect fauna as assessed by Hörren et al. (2022) based on 34,085 insect species (38% herbivores, 16% predators, 27% parasitoids, 19% detritivores). In addition to functional groups, we separately analyzed four large taxonomic orders of flying insects (Lepidoptera, Coleoptera, Hymenoptera, Diptera), covering 87% of all BINs. For an overview of BIN numbers within functional and taxonomic arthropod groups included in the analyses, see Table S2. For details on arthropod sampling, metabarcoding and bioinformatics, see Uhler et al. (2021).

### Vegetation survey

Vegetation data were collected during summer 2019 and 2020, between late May and late July in both years. In the first year, species composition of vascular plants (hereafter referred to as ‘plants’) was recorded within seven subplots of 1.2 m × 1.2 m size (10 m^2^ in total) distributed over each study site (3 m × 30 m). In the second year, plant species pools were assessed within a 200-m buffer area around each Malaise trap. Species were recorded by standardized walks along transects that led along existing roads or field paths across the buffer area, and covered the prevailing habitat types surrounding the traps. Walking time was proportional to the area percentages of habitat types present within the 200-m radius and standardized to 60 min in total. On average, 75% of all plant species observed at the 3 m × 30 m study site were recorded during the corresponding transect walk. We combined the data from both surveys in one presence–absence matrix for further analysis to include the most comprehensive information on the plant species pools in close proximity and within the further surroundings of the trap location, given that arthropods, depending on their mobility, may have reached the Malaise traps from various distances. The combined set of plant species reflected differences and gradients between habitat types more clearly than the plant species assessed on the 3 m × 30 m study sites, which, due to site selection standardization (open herbaceous vegetation), were more uniform in their vegetation (Fig. S1a, b).

### Environmental data

We used a detailed land-cover map including combined information from IACS 2019 (Integrated Administration and Control System), ATKIS 2019 (German Official Topographic-Cartographic Information System), and CORINE land-cover data (Coordination of information on the environment; CLMS 2018) to calculate land-cover composition (i.e., percentages of land-cover classes present within a 200-m buffer around the study sites); see Kandziora et al. (2013) for detailed descriptions of all three data sources. We differentiated the following 12 land-cover classes: annual crop, perennial crop, managed grassland, succession area, small woody features, coniferous, deciduous and mixed forest, wetland, water, roads, and settlement areas. Choosing the 200-m radius allowed us to compare the effects of plant species composition and land cover at the same spatial scale. Land-cover composition in the 200-m buffer areas corresponded to the focal habitat types of the study sites (forest, grassland, arable field, settlement; Fig. S1c) and at the same time, represented gradients of local land use more accurately than the four habitat categories.

We retrieved multi-annual mean temperature and precipitation values for the study sites on a monthly basis and at a spatial resolution of 1 km × 1 km from the German Meteorological Service (DWD Climate Data Center 2021a, b) to calculate mean summer temperature and total summer precipitation (May–September) as well as mean annual temperature range (difference between mean January and July temperature) for the period 1991–2020. In addition, we included elevation of the sites in the climatic predictor set, using a digital terrain model with a spatial resolution of 30 m (Earth Resources Observation and Science Center 2017). To control for effects of spatial autocorrelation, we included longitude and latitude of the study sites (hereafter referred to as ‘space’) in the analysis.

### Statistical analyses

We conducted all statistical analyses in R 4.0.3 (R Core Team 2020). For each arthropod group, we created a presence–absence matrix aggregating the total lists of BINs per study site across all three sampling periods. To represent differences in arthropod taxonomic composition as response variables, we calculated between-site dissimilarity matrices for each arthropod group using the distance version of the Sørensen coefficient (Sørensen 1948; Legendre and Legendre 2012). Accordingly, we calculated dissimilarity matrices of the predictor sets using Euclidean distances for land-cover composition, climate and space, and Sørensen distances for plant species composition. Since the climatic predictor set contained variables measured on different scales, we standardized these variables by dividing them by their maximum value prior to calculation of Euclidean distances. The dissimilarity matrix for plant species composition was derived from the presence-absence matrix based on the combined data from the vegetation assessment on the study sites and the transect walks within 200-m radius of the sites.

Correlations between all predictor matrices were assessed using Pearson correlation coefficients. Mantel tests were used to calculate coefficients and p-values (function *mantel* in *vegan*; Oksanen et al. 2020) (Fig. S2). To assess independent and shared explanatory contributions of the predictors, we used variation partitioning based on distance-based redundancy analysis (function *varpart* in *vegan*; Oksanen et al. 2020). As this method allows for collinearity within and between predictor matrices (Oksanen et al. 2020), it is a suitable tool for evaluating the combined effects of two or more predictors. To assess differences in the influence of plant species composition among trophic levels, we compared the independent explanatory contribution of plant species composition as obtained from variation partitioning between the respective groups.

We used Ellenberg indicator values as an additional predictor to compare the effects of plant species composition and local abiotic conditions on arthropod assemblage composition. These indicator values were developed to quantify the environmental conditions experienced by plant communities of the Central European flora, while their applicability extends to other parts of Europe (see Diekmann 2003 for an overview). Based on ordinal scales ranging from 1 to 9, the values indicate under which conditions a species is most likely to occur (Ellenberg et al. 1991). Since they are closely correlated to corresponding environmental measurements (e.g., Schaffers and Sýkora 2000; Reger et al. 2011) and show remarkable robustness against incomplete floristic data (Ewald 2003), Ellenberg indicator values are a suitable proxy for measuring habitat conditions. We retrieved the values for the sampled plant species from the German Species List (Jansen and Dengler 2008; https://germansl.infinitenature.org/) and created an attribute matrix containing relative frequencies for all classes of indicator plants of light, temperature, continentality, moisture, soil pH, and nutrients (Schmidtlein and Ewald 2003). Species without indicator values —as was the case for exotic species or species showing indifferent responses to the respective conditions—were excluded from the calculation. The proportion of plant species to which indicator values could be assigned varied among the classes and ranged between 54 and 71% for temperature and light, respectively (Table S3). Based on the obtained matrix, we calculated Euclidean distances between the sites. We then assessed independent and shared effects of plant species composition and indicator values on arthropod taxonomic composition using variation partitioning as described above. To explore what kind of environmental indicator information is most relevant, we correlated arthropod dissimilarities with Euclidean distances of each single indicator value using Mantel tests.

To evaluate the explanatory contribution of trophic links between plants and arthropods, we created a list of monophagous herbivores based on the metabarcoding species identifications. Only species that were identified with 97% probability or more were included in this analysis. We considered monophagous herbivores as those feeding on one plant genus (Cates 1980; Müller et al. 2011), according to available literature. From the recorded plant species, we filtered those species belonging to host plant genera of monophagous herbivores. We then performed another version of variation partitioning, considering only this subset of host plants along with Ellenberg indicator values. To assess the significance of the host plants for arthropod assemblages, we repeated this step 1000 times, each time using a randomly selected plant subset of the same size as the host plant subset. Using *t*-tests, we evaluated if the explanatory contribution of the subset of host plants differed from the average explanatory contribution of the randomly selected plant subsets.

## Results

Total BIN numbers per site varied between 438 and 1247 (Table S4). Overall, 3535 arthropod species could be determined with 97% probability, containing 1906 herbivores of which 400 (21%) were monophagous (i.e., feeding on one plant genus). In total, we recorded 987 plant species from 114 families. Plant species numbers per site varied between 52 and 204 (Table S4). Of all observed plant species, 50% belonged to the host plant genera of the 400 monophagous herbivores in our dataset. For information on the distribution of (host) plant species across families, see Table S5. For 76 ± 12% (mean ± SD) of the monophagous herbivores occurring at a study site, we recorded the matching host plant genus within the 200-m buffer area of that site.

**Fig. 2:**
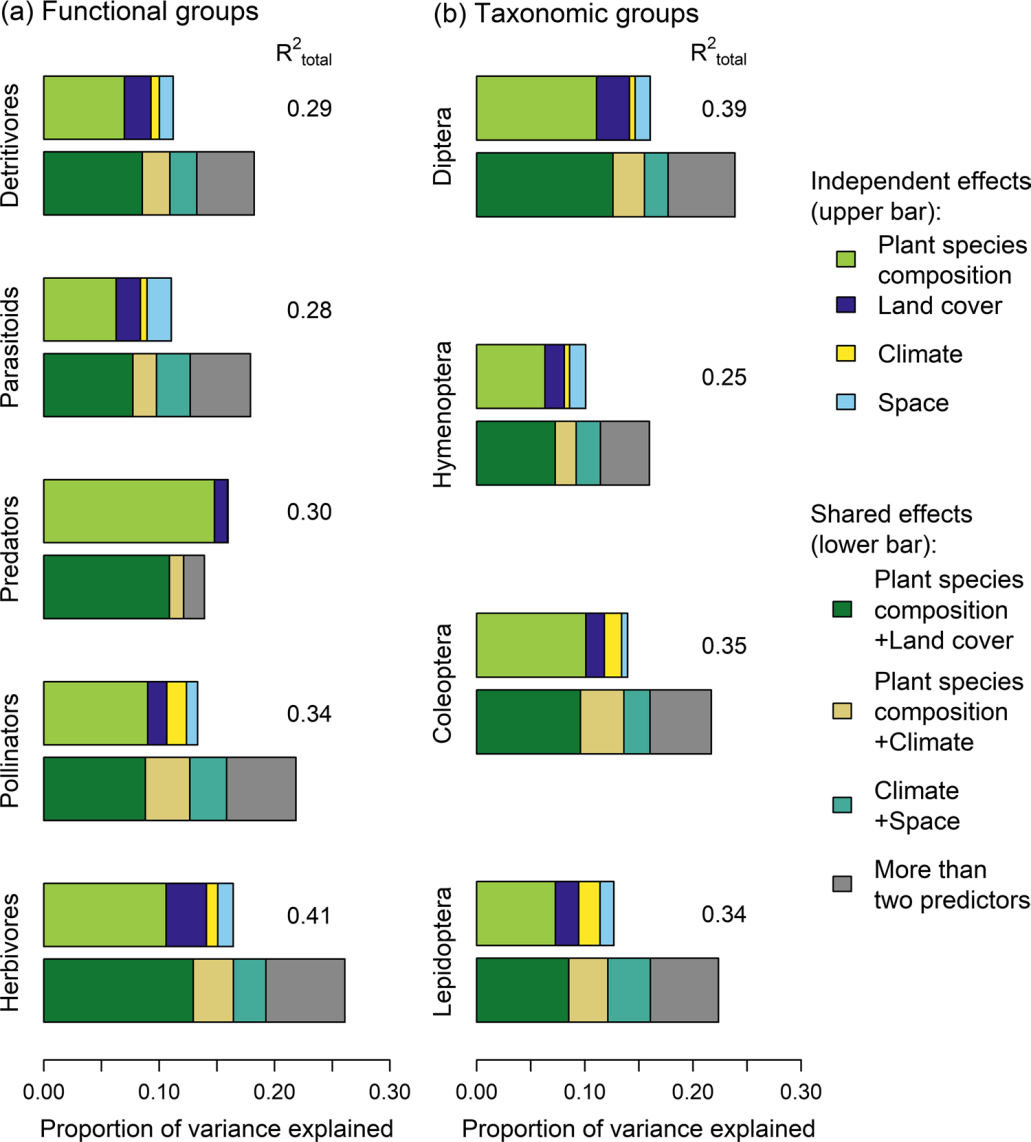
Proportion of variance explained (adjusted *R*^2^) in the assemblage composition of five functional (**a**) and four taxonomic arthropod groups (**b**). Two bars are shown for each group: the upper bar indicates independent proportions of variance explained by plant species composition, land-cover composition, climate and space. The lower bar indicates proportions of variance explained by the combination of two (colored segments) or more predictors (grey segment). Total adjusted *R*^2^-values are shown next to the bars. *R*^2^-values of single fractions are given in Tables S6. Arthropod dissimilarities between sites were calculated using the Sørensen index. Predictive dissimilarity matrices were calculated using the Sørensen index (plant species composition) and Euclidean distances (land cover, climate and space)

Across all investigated groups, the largest fractions of variance in arthropod taxonomic composition were explained by the independent effect of plant species composition and the shared effect of plant species composition and land cover (Fig. 2). Both of these fractions explained similar amounts of variance in each group, while the independent effect of land cover accounted only for a minor fraction of variance (< 4% in each group). Climate and space did not substantially contribute to explained variance. Total variance explained by plant species composition, land cover, climate, and space ranged from 29% (detritivores) to 41% (herbivores) among functional groups and from 25% (Hymenoptera) to 39% (Diptera) among taxonomic groups (Fig. 2). All R^2^-values are given in Tables S6.

Variance explained by the independent effect of plant species composition showed no distinct decrease with trophic levels (Fig. 2). Plant species composition was most important at the intermediate trophic level (predators). In combination with land-cover composition, it accounted for almost the entire explained variance in predator assemblages. Responses of herbivore and pollinator assemblages to plant species composition were less strong than those of predators, but stronger than those of parasitoids and detritivores. Moreover, herbivores showed a stronger response to the combined effect of plant species composition and land-cover composition than to the independent effect of plant species composition (Table S6).

**Fig. 3:**
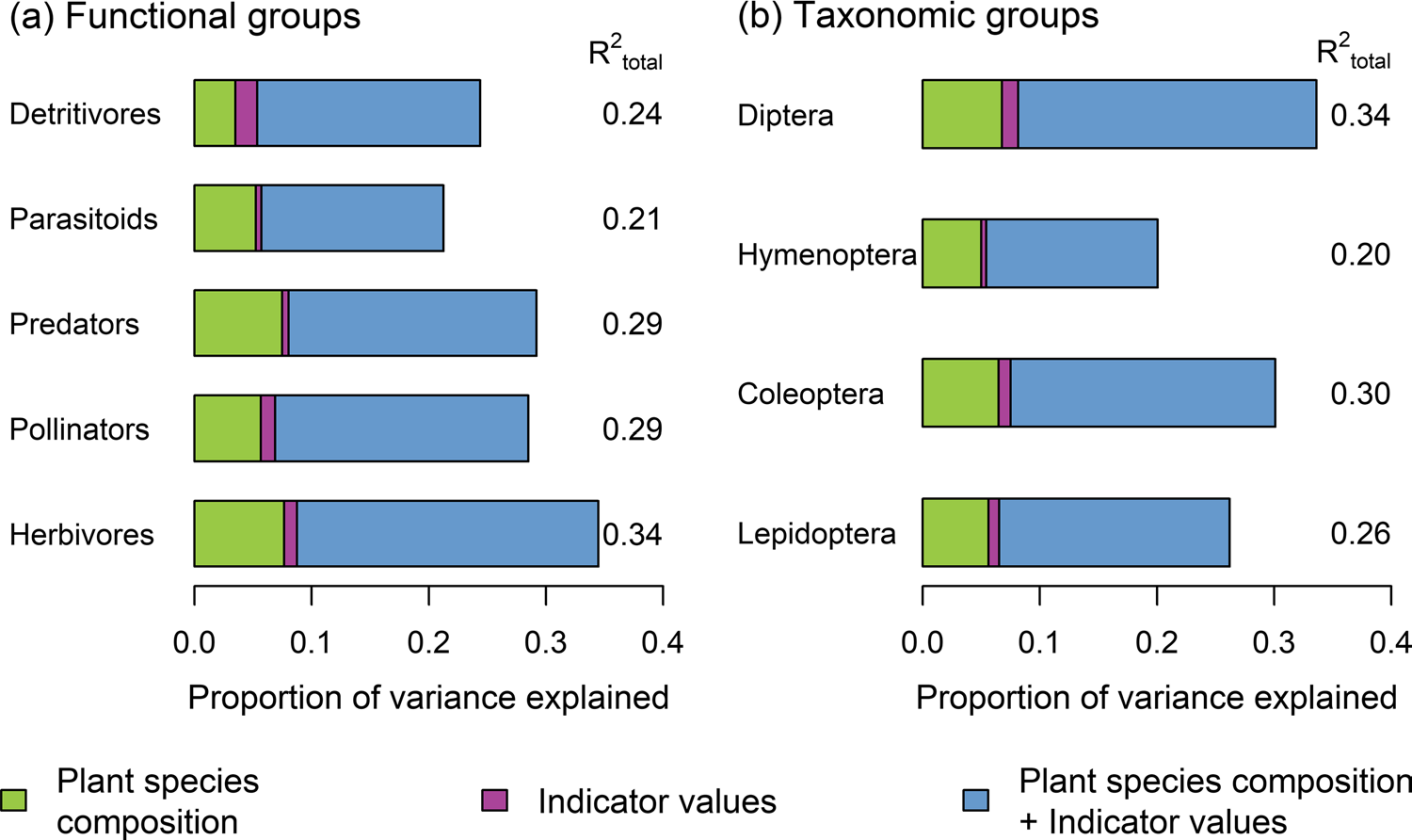
Proportion of variance explained (adjusted *R*^2^) in the assemblage composition of five functional (**a**) and four taxonomic arthropod groups (**b**). Bar segments indicate independent and shared effects of plant species composition and Ellenberg indicator values. Arthropod dissimilarities were calculated using the Sørensen index. Predictive dissimilarity matrices were calculated using the Sørensen index (plant species composition) and Euclidean distances (indicator values)

Comparing the direct effect of plant species composition and local abiotic conditions measured by Ellenberg indicator values revealed that the largest fraction of vegetation-related variance was explained by the shared effect of plant species composition and indicator values (Fig. 3). This was consistent across all taxonomic and functional groups. This means that, if only the simplified dataset of Ellenberg indicator values and no information on plant species identity were included in the analysis, then indicator values would still account for the major fraction of variance in arthropod taxonomic composition (Table S7).

**Fig. 4:**
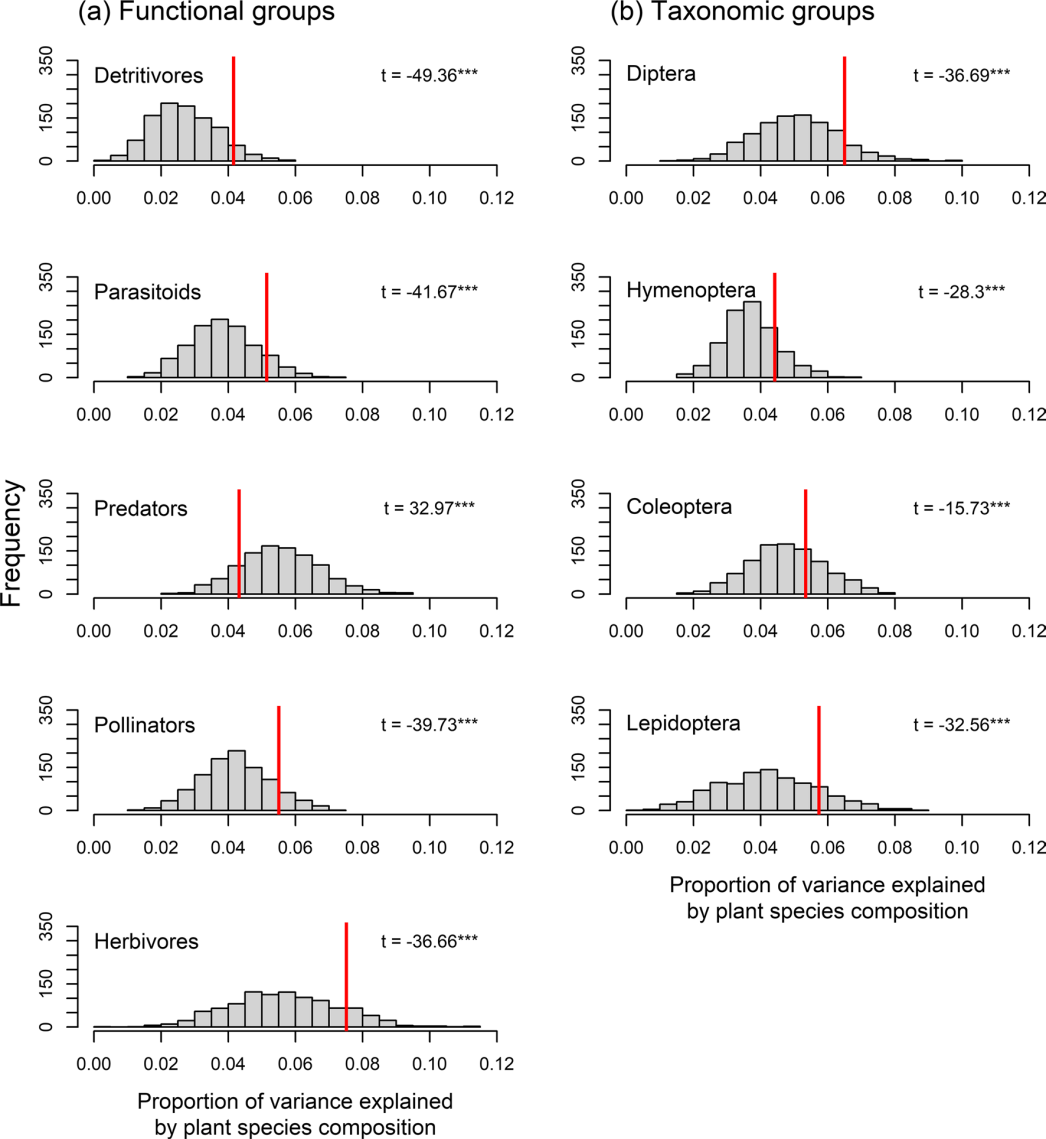
Histograms and t-values (significance levels: ***p < 0.001, **p < 0.01, *p < 0.05) comparing independent proportions of variance explained (adjusted *R*^2^) in the assemblage composition of five functional (**a**) and four taxonomic arthropod groups (**b**). Red lines indicate variance explained by plant species composition using a subset of host plants for monophagous herbivores, compared to 1000 randomly selected subsets of equal size (grey bars)

Repeating variation partitioning, as shown in Fig. 3, with a dataset reduced to include only host plants of monophagous herbivores led to similar amounts of explained variance in the assemblages of all functional groups except for predators, which responded best to the full set of plant species (Table 1). Furthermore, t-tests showed that the subset of host plants performed significantly better in explaining arthropod composition than the randomly selected plant subsets of equal size for all taxonomic orders and almost all functional groups (Fig. 4). Only in predator assemblages, the subset of host plants explained significantly less variance than randomly selected subsets of plant species.

**Table 1:** Independent explained variance of plant species composition in arthropod assemblage composition including all plant species, a subset of only those plant species belonging to host plant genera of monophagous herbivores, and equally sized subsets of randomly selected plant species

	All plant species (*n* = 987)	Only host plants (*n* = 504)	Mean ± SD of 1000 random selections (*n* = 504)
Detritivores	0.035	0.042	0.027 ± 0.009
Parasitoids	0.052	0.051	0.039 ± 0.010
Predators	0.075	0.043	0.056 ± 0.012
Pollinators	0.057	0.055	0.042 ± 0.010
Herbivores	0.077	0.075	0.057 ± 0.016
Diptera	0.068	0.065	0.051 ± 0.012
Hymenoptera	0.050	0.044	0.037 ± 0.008
Coleoptera	0.065	0.053	0.048 ± 0.011
Lepidoptera	0.056	0.057	0.042 ± 0.015

Adjusted *R*^2^-values are shown

Separating between different categories of Ellenberg indicator values, temperature values showed the highest correlations with arthropod assemblage composition in most groups (Table 2). Pearson coefficients for light values were similarly high in most groups and exceeded those for temperature values in parasitoids. For predators, soil moisture was the second most correlated indicator value. Across all investigated groups, correlations were most pronounced when including the full set of indicator values.

**Table 2:** Correlations between arthropod assemblage composition and categories of Ellenberg indicator values

	All values	Light	Tempera-ture	Moisture	Soil pH	Nutrients	Conti-nentality
Detritivores	0.46	0.36	**0.38**	0.28	0.35	0.22	0.21
Parasitoids	0.40	**0.35**	0.32	0.25	0.26	0.18	0.25
Predators	0.47	0.34	**0.39**	0.37	0.35	0.15	0.24
Pollinators	0.48	0.35	**0.38**	0.33	0.33	0.25	0.26
Herbivores	0.52	0.41	**0.42**	0.34	0.35	0.26	0.27
Diptera	0.52	0.41	**0.44**	0.34	0.37	0.24	0.25
Hymenoptera	0.39	**0.34**	0.31	0.24	0.24	0.18	0.26
Coleoptera	0.49	0.36	**0.39**	0.31	0.36	0.27	0.27
Lepidoptera	0.45	0.34	**0.36**	0.32	0.32	0.24	0.21

Pearson coefficients were calculated using Sørensen dissimilarities for arthropod groups and Euclidean distances for Ellenberg indicator values. Bold values show highest coefficients within single categories of indicator values

## Discussion

In this study, we investigated independent and shared effects of plant species composition and abiotic drivers on terrestrial arthropod assemblage composition across different taxonomic orders and functional groups. Plant species composition was the primary determinant of arthropod composition but also shared a substantial fraction of explained variance with land-cover composition. Moreover, the local habitat conditions depicted by the indicator values of the plants grown at the sites were more important for arthropod composition than trophic relationships between certain plant and arthropod species. The explanatory power of plant species composition for arthropod composition did not decrease uniformly toward higher trophic levels, but was strongest for predators.

The largest part of the variance in the composition of arthropods across all taxonomic orders and functional groups explained by the studied variables was due to variation in plant species composition. In each group, about half of this fraction was shared by the effect of land-cover composition. This overlap is not surprising since land use determines vegetation composition and structure in space and time (Knapp et al. 2010; Börschig et al. 2013; Deguines et al. 2016). Moreover, it shows that the explanatory contribution of vegetation can partly be attributed to general habitat characteristics summarized by land-cover classes. Previous studies showed that information on overall habitat type may often be sufficient to explain differences in arthropod assemblages, as demonstrated for Coleoptera (Blake et al. 2003) and butterflies (Bergerot et al. 2011). Schaffers et al. (2008) found that the predictive power of vegetation composition for arthropod species composition was independent of how detailed the vegetation was assessed (individual plant species, characteristic species groups, and plant community type). In our case, a substantial fraction of variance was due to the independent effect of plant species composition (Fig. 2), indicating that both general information on habitat type as well as detailed information at the plant species level are relevant and complementary predictors of arthropod assemblage composition.

Primary consumers are assumed to be more strongly influenced by plant species composition than higher trophic groups (Sanderson et al. 1995; Stoner and Joern 2004; Bae et al. 2021). In our study, plant species composition explained less variance of the assemblage composition of parasitoids and detritivores than of herbivores and pollinators. However, predator assemblage composition showed the strongest response to the independent effect of plant species composition, compared to the other trophic groups. Strong associations between plant species composition and predator assemblages have also been reported for spiders and ground beetles (Blake et al. 2003; Beals et al. 2006; Schaffers et al. 2008). Possible explanations may be bottom-up effects of plant communities propagating through food webs (Scherber et al. 2010), responses to physiognomic plant traits reflecting fine-scale habitat architecture (Halaj et al. 2000; Beals 2006), or edaphic factors to which both plant and arthropod communities respond (Blake et al. 2003). Regardless of the underlying mechanisms, our results show that the strong predictive power of plant species composition for arthropod composition is not limited to phytophages or ground-dwellers only but also extends to a broad taxonomic range of predators.

When studying relationships between plant and arthropod communities, a major challenge is to distinguish between direct biotic interactions and indirect abiotic effects that are mediated by vegetation characteristics (Schaffers et al. 2008; Müller et al. 2011), but hard to detect by physico-chemical measurements (Schaffers et al. 2008; Zellweger et al. 2017). Our approach was to quantify these aspects by comparing the explanatory contributions of plant species composition and Ellenberg indicator values (Fig. 3). In all groups, we found that the shared effect of both predictors explained the major fraction of variance. This may seem obvious since Ellenberg indicator values are derived from the plant species data and are thus strongly correlated with plant species composition (Fig. S2). However, it also shows that a large fraction of vegetation-related variance can be explained using a greatly simplified plant dataset (i.e., reduced to Ellenberg indicator values) that summarizes information on environmental gradients, but does not contain information on single species. Local abiotic conditions may thus be more relevant in shaping arthropod assemblages than the occurrence of certain plant species (Müller et al. 2011; van Schalkwyk et al. 2019). Thus, floristic datasets provide key ecological information to characterize habitat niches of arthropods in an accurate way (Schaffers et al. 2008).

Beyond these prevailing relationships, we were interested in the remaining independent effect of plant species composition on arthropods after accounting for local abiotic conditions. For herbivores, pollinators and all taxonomic orders, the subset of host plants of monophagous herbivores performed better in explaining arthropod composition than randomly selected combinations of plant species. This suggests that direct trophic links between plants and arthropods account for the part of variance independently explained by plant species composition. Surprisingly, we also observed this effect for parasitoids and detritivores (Fig. 4). Traits of host plants of monophagous herbivores may possibly overlap with other traits that are relevant to multiple trophic groups. Moreover, trophic interactions between specialized insects and their host plants may be obscured by the neighboring plant community (Kostenko et al. 2017). Host specialization often coincides with habitat specialization since specialized insects are unlikely to occupy the entire range of their host plants (Quinn et al. 1998; Bogusch et al. 2020). Müller et al. (2011) demonstrated that plant species composition had high predictive power for the species composition of phytophagous specialists, but this effect was not due to the occurrence of specific host plants. These findings do not deny the importance of host plants for specialized insects. Instead, they support the view that specialized herbivores probably first inspect suitable habitat conditions before searching for their host plant (Müller et al. 2011).

The subset of plants known to be host plants for monophagous herbivores explained significantly less variance in predator assemblages than randomly selected subsets of plant species. Given that predators tend to be more opportunistic regarding their prey species than e.g., parasitoids (Strand and Obrycki 1996; Price 2020), their response to plant species composition may be independent of trophic mechanisms mediated by the herbivore community. Instead, structural characteristics of vegetation may be a more relevant aspect in determining predator communities (Brose 2003; Langellotto and Denno 2004). This matches our finding that the complete set of plant species clearly performed better in predicting predator composition than any subset of plant species, given that each recorded plant species adds more information on the fine-scale structural complexity of a habitat.

Comparing the effect of single abiotic conditions, we found that temperature and light indicator values were most strongly correlated with the assemblage composition of all arthropod groups. In contrast, long-term temperature data with a spatial resolution of 1 km × 1 km explained very low amounts of variation. Arthropod community composition is known to respond to long-term climate (Zellweger et al. 2017; Uhey et al. 2020) as well as to local-scale temperatures (Prather and Kaspari 2019; Uhler et al. 2021). Plant species composition summarizes fine-scale microclimatic patterns for any desired area (here, 200-m buffer areas around the Malaise traps) and worked as a better proxy of local climatic conditions in our case. Small-scale temperature and light availability are both sensitive to major drivers of global change, with habitats experiencing higher temperatures as well as increases in vegetation height and density due to climate warming and land-use change (Rautiainen et al. 2011; Govaert et al. 2021). Our results indicate that changes in these habitat variables may cause shifts in arthropod assemblage composition. Yet, we point out that the complete set of plant indicator values still showed the strongest correlations with arthropod composition, suggesting that a range of habitat conditions predicts arthropod assemblages more accurately than single environmental gradients. In the context of biodiversity monitoring, vegetation assessments can thus provide valuable information to assess the habitat suitability for arthropods, particularly regarding first- and second-order consumers. Therefore, we emphasize the usefulness to consider plant species composition as a proxy for measuring habitat conditions that, due to high short-term variation, effort and cost, are hard to assess by direct environmental measurements (Diekmann 2003). While plant indicator values are available for several European countries (Diekmann 2003), such frameworks still need to be developed for other continents.

Overall variance explained by the predictors included in the analyses ranged between 25 and 41%, raising the question of which factors could account for those fractions of variance that remained unexplained. For example, we did not consider potential differences in land-use intensity within the studied habitat types, such as the use of pesticides or fertilizer. These factors are known to affect arthropod composition (e.g., Börschig et al. 2013; Sohlström et al. 2022), and would likely have contributed additional explained variance to our analyses, albeit such data was not available for our study sites. Moreover, we did not account for differences in weather conditions among the study sites throughout the sampling season. Including this information would probably have added explained variance, since local weather conditions are a strong driver of arthropod populations (Hausmann et al. 2022) and thus affect species composition. Despite these restrictions, including a wide variety of sampling locations spread across a large spatial extent, our results provide valuable insights into the drivers of arthropod community composition in cultural landscapes of Central Europe.

Extending the findings of Schaffers et al. (2008), we identified plant species composition as the main determinant of arthropod assemblage composition across multiple taxa and trophic levels in the most common habitats of temperate landscapes. Overall differences in habitat type, as well as fine-scale abiotic gradients reflected by vegetation, mainly contributed to the predictive power of plant species composition, while the occurrence of certain (host) plant species was subordinated to these environmental filters. Our results highlight the importance of considering both direct and integrative aspects of vegetation when examining the relationships between plant and arthropod communities.

## Supplementary Information

Below is the link to the electronic supplementary material.

Supplementary file1 (DOCX 403 KB)

## Data Availability

The datasets analyzed during the study are available in the Figshare Repository at 10.6084/m9.figshare.16860025.

## Code availability

R Code is available from the corresponding author on reasonable request.
