# Supplementary material for: Plant species composition and local habitat conditions as primary determinants of terrestrial arthropod assemblages
Source: Oecologia. 2023 Mar 3;201(3):813–25. doi: 10.1007/s00442-023-05345-6 (PMC10038969; doi:10.1007/s00442-023-05345-6)
Supplement: Supplementary file 1 — Supplementary file1 (DOCX 403 KB) [file 442_2023_5345_MOESM1_ESM.docx]

**Electronic supplementary material**

Plant species composition and local habitat conditions as primary determinants of terrestrial arthropod assemblages

Cynthia Tobisch^1,2^*, Sandra Rojas-Botero^2^, Johannes Uhler^3^, Jörg Müller^3,4^, Johannes Kollmann^2^, Christoph Moning^1^, Martin Brändle^5^, Martin M. Gossner^6,7^, Sarah Redlich^8^, Jie Zhang^8^, Ingolf Steffan-Dewenter^8^, Caryl Benjamin^9^, Jana Englmeier^3^, Ute Fricke^8^, Cristina Ganuza^8^, Maria Haensel^10^, Rebekka Riebl^10^, Lars Uphus^9^, Jörg Ewald^1^

^1^Institute of Ecology and Landscape, Weihenstephan-Triesdorf University of Applied Sciences, Freising, Germany. ^2^Chair of Restoration Ecology, School of Life Sciences, Technical University of Munich, Freising, Germany. ^3^Field Station Fabrikschleichach, Department of Animal Ecology and Tropical Biology, Julius-Maximilians-University Würzburg, Würzburg, Germany. ^4^Bavarian Forest National Park, Grafenau, Germany. ^5^Division of Animal Ecology, Department of Ecology, Philipps-Universität Marburg, Marburg, Germany. ^6^Forest Entomology, Swiss Federal Institute for Forest, Snow, and Landscape Research WSL, Birmensdorf, Switzerland. ^7^Department of Environmental Systems Science, Institute of Terrestrial Ecosystems, ETH Zürich, Zürich, Switzerland. ^8^Department of Animal Ecology and Tropical Biology, Julius-Maximilians-University Würzburg, Würzburg, Germany. ^9^Ecoclimatology, School of Life Sciences, Technical University of Munich, Freising, Germany. ^10^Professorship of Ecological Services, Bayreuth Center of Ecology and Environmental Research (BayCEER), University of Bayreuth, Bayreuth, Germany.

*corresponding author: [cynthia.tobisch@hswt.de](mailto:cynthia.tobisch@hswt.de)

[Fig. S1 2](#_Toc127539358)

[Fig. S2 3](#_Toc127539359)

[Table S1 4](#_Toc127539360)

[Table S2 11](#_Toc127539361)

[Table S3 12](#_Toc127539362)

[Table S4 13](#_Toc127539363)

[Table S5 14](#_Toc127539364)

[Table S6 15](#_Toc127539365)

[Table S7 16](#_Toc127539366)

**
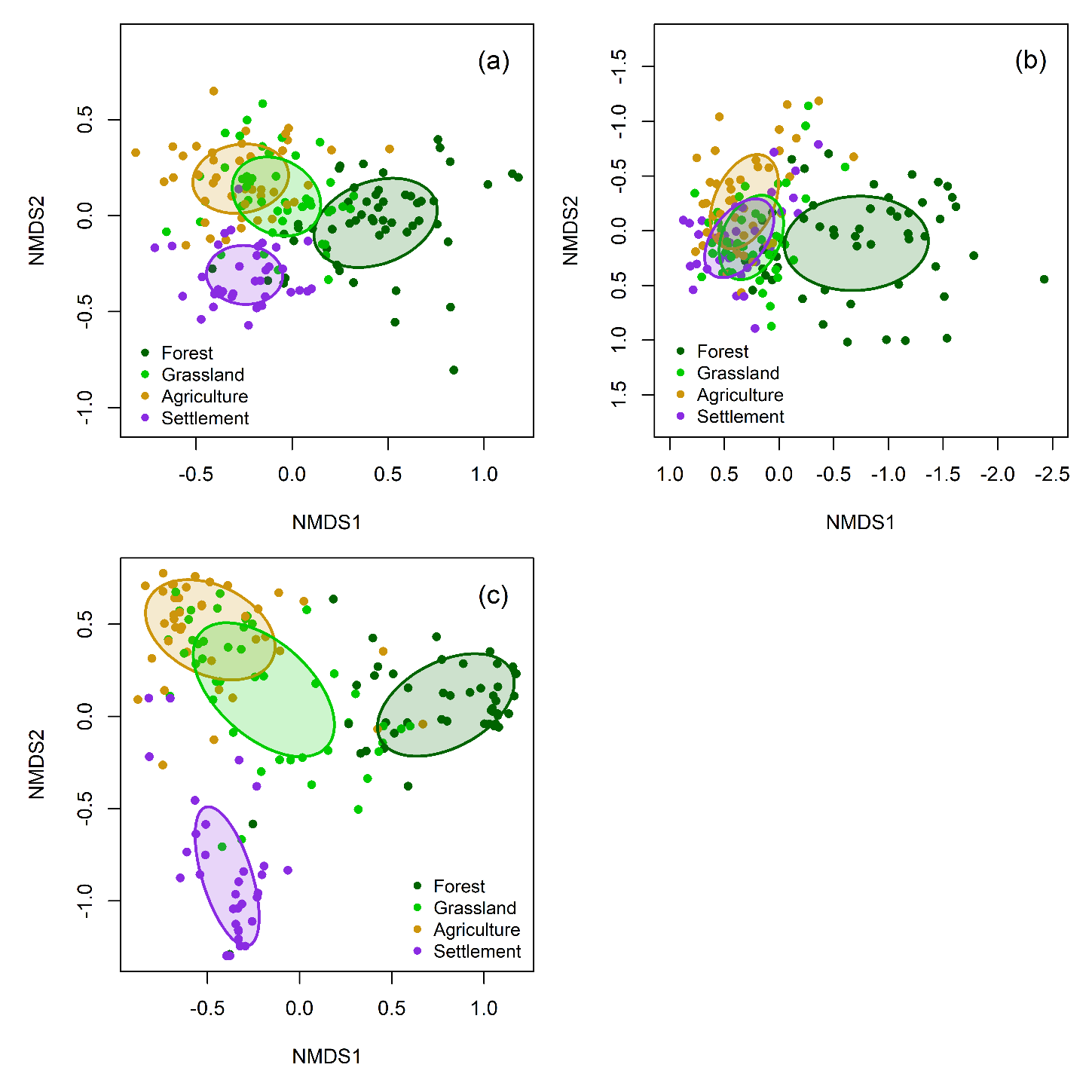
**

Fig. S1 Ordination plots based on non-metric multidimensional scaling (NMDS) of (a) plant species composition assessed within 200-m radius of the study sites (including the species recorded directly on the sites), (b) plant species composition assessed directly on the study sites, and (c) land cover composition in 200-m radius of the sites. Sørensen index was used as a dissimilarity measure for plant species composition and Bray-Curtis index for land-cover composition. Different colors show the habitat types of the study sites. Ellipses delimit the standard deviation of points from the center of the point cloud of the respective habitat. Each NMDS was calculated using four dimensions, with stress values of 0.119 (a), 0.134 (b), and 0.067 (c).


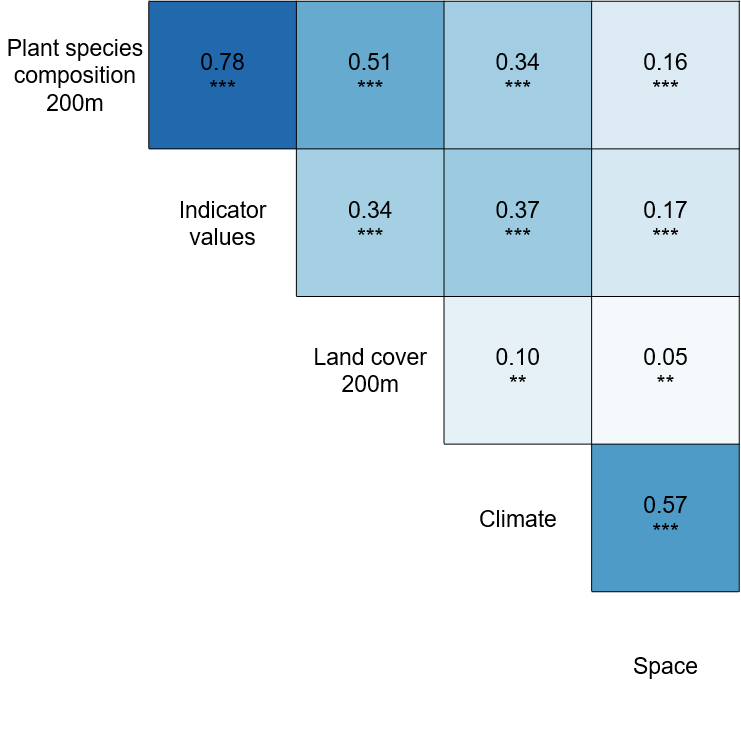


Fig. S2 Correlation matrix of environmental predictors; Pearson coefficients were calculated for between-site dissimilarities of (i) plant species composition within 200 m radius of the study sites (including the species recorded directly on the sites), (ii) relative proportions of Ellenberg indicator values of the plant species occurring within 200 m radius of the sites, (iii) land-cover composition within 200 m radius of the study sites, (iv) climate variables (elevation, multi-annual mean summer temperature, total summer precipitation, mean annual temperature range for the period 1991–2020), and (v) spatial distribution (x- and y-coordinates) of the sites, using Sørensen dissimilarities for plant species composition and Euclidean distances for all other predictors. Asterisks indicate significance levels of p-values calculated by Mantel-tests (*** p < 0.001, ** p < 0.01, * p < 0.05).

Table S1 List of all arthropod orders and families included in the analysis as well as their assignment to functional groups

| **Order** | **Family** | **Herbi-vore** | **Polli-nator** | **Preda-tor** | **Para-site** | **Para-sitoid** | **Detri-tivore** | **None** |
| --- | --- | --- | --- | --- | --- | --- | --- | --- |
| Araneae |  |  |  | x |  |  |  |  |
| Araneae | Agelenidae |  |  | x |  |  |  |  |
| Araneae | Araneidae |  |  | x |  |  |  |  |
| Araneae | Clubionidae |  |  | x |  |  |  |  |
| Araneae | Dictynidae |  |  | x |  |  |  |  |
| Araneae | Gnaphosidae |  |  | x |  |  |  |  |
| Araneae | Linyphiidae |  |  | x |  |  |  |  |
| Araneae | Liocranidae |  |  | x |  |  |  |  |
| Araneae | Lycosidae |  |  | x |  |  |  |  |
| Araneae | Philodromidae |  |  | x |  |  |  |  |
| Araneae | Pisauridae |  |  | x |  |  |  |  |
| Araneae | Salticidae |  |  | x |  |  |  |  |
| Araneae | Sparassidae |  |  | x |  |  |  |  |
| Araneae | Tetragnathidae |  |  | x |  |  |  |  |
| Araneae | Theridiidae |  |  | x |  |  |  |  |
| Araneae | Thomisidae |  |  | x |  |  |  |  |
| Coleoptera |  |  |  |  |  |  |  | x |
| Coleoptera | Aderidae |  |  |  |  |  |  | x |
| Coleoptera | Anthicidae |  |  |  |  |  |  | x |
| Coleoptera | Anthribidae |  |  |  |  |  | x |  |
| Coleoptera | Attelabidae |  |  |  |  |  |  | x |
| Coleoptera | Bostrichidae |  |  |  |  |  |  | x |
| Coleoptera | Brentidae |  |  |  |  |  |  | x |
| Coleoptera | Buprestidae | x | x |  |  |  |  | x |
| Coleoptera | Byrrhidae | x |  |  |  |  |  |  |
| Coleoptera | Byturidae | x |  |  |  |  |  |  |
| Coleoptera | Cantharidae |  |  | x |  |  |  |  |
| Coleoptera | Carabidae |  |  | x |  |  |  |  |
| Coleoptera | Cerambycidae | x |  |  |  |  |  |  |
| Coleoptera | Chrysomelidae | x |  |  |  |  |  |  |
| Coleoptera | Ciidae |  |  |  |  |  |  | x |
| Coleoptera | Cimberididae |  |  |  |  |  |  | x |
| Coleoptera | Cleridae |  |  | x |  |  |  |  |
| Coleoptera | Coccinellidae |  |  | x |  |  |  |  |
| Coleoptera | Corylophidae |  |  |  |  |  |  | x |
| Coleoptera | Cryptophagidae |  |  |  |  |  | x |  |
| Coleoptera | Curculionidae | x |  |  |  |  |  |  |
| Coleoptera | Dascillidae |  |  |  |  |  |  | x |
| Coleoptera | Dermestidae |  |  |  |  |  |  | x |
| Coleoptera | Dytiscidae |  |  |  |  |  |  | x |
| Coleoptera | Elateridae |  |  |  |  |  |  | x |
| Coleoptera | Elmidae |  |  |  |  |  |  | x |
| Coleoptera | Erotylidae |  |  |  |  |  |  | x |
| Coleoptera | Eucnemidae |  |  |  |  |  |  | x |
| Coleoptera | Geotrupidae |  |  |  |  |  | x | x |
| Coleoptera | Haliplidae |  |  |  |  |  |  | x |
| Coleoptera | Helophoridae |  |  |  |  |  |  | x |
| Coleoptera | Hydrophilidae |  |  |  |  |  |  | x |
| Coleoptera | Kateretidae | x |  |  |  |  |  |  |
| Coleoptera | Lampyridae |  |  | x |  |  |  |  |
| Coleoptera | Latridiidae |  |  |  |  |  | x |  |
| Coleoptera | Leiodidae |  |  |  |  |  | x |  |
| Coleoptera | Lucanidae |  |  |  |  |  |  | x |
| Coleoptera | Lycidae |  |  | x |  |  |  |  |
| Coleoptera | Lymexylidae |  |  |  |  |  |  | x |
| Coleoptera | Melandryidae |  |  |  |  |  | x |  |
| Coleoptera | Meloidae |  |  |  |  |  |  | x |
| Coleoptera | Melyridae |  |  |  |  |  |  | x |
| Coleoptera | Monotomidae |  |  |  |  |  |  | x |
| Coleoptera | Mordellidae |  |  |  |  |  | x |  |
| Coleoptera | Mycetophagidae |  |  |  |  |  |  | x |
| Coleoptera | Nitidulidae |  |  |  |  |  |  | x |
| Coleoptera | Oedemeridae | x |  |  |  |  |  |  |
| Coleoptera | Omalisidae |  |  |  |  |  |  | x |
| Coleoptera | Peltidae |  |  |  |  |  |  | x |
| Coleoptera | Phalacridae |  |  |  |  |  |  | x |
| Coleoptera | Ptinidae |  |  |  |  |  | x |  |
| Coleoptera | Pyrochroidae |  |  |  |  |  |  | x |
| Coleoptera | Rhadalidae |  |  |  |  |  |  | x |
| Coleoptera | Salpingidae |  |  |  |  |  |  | x |
| Coleoptera | Scarabaeidae |  |  |  |  |  |  | x |
| Coleoptera | Scirtidae |  |  |  |  |  | x |  |
| Coleoptera | Scraptiidae | x |  |  |  |  |  |  |
| Coleoptera | Silphidae |  |  |  |  |  | x |  |
| Coleoptera | Silvanidae |  |  |  |  |  | x |  |
| Coleoptera | Sphindidae | x |  |  |  |  |  |  |
| Coleoptera | Staphylinidae |  |  | x |  |  |  |  |
| Coleoptera | Tenebrionidae |  |  |  |  |  | x |  |
| Coleoptera | Tetratomidae | x |  |  |  |  |  |  |
| Coleoptera | Throscidae | x |  |  |  |  |  |  |
| Diptera |  |  |  |  |  |  |  | x |
| Diptera | Acartophthalmidae |  |  |  |  |  |  | x |
| Diptera | Acroceridae |  |  |  |  |  |  | x |
| Diptera | Agromyzidae | x |  |  |  |  |  |  |
| Diptera | Anisopodidae |  |  |  |  |  | x |  |
| Diptera | Anthomyiidae | x |  |  |  |  |  |  |
| Diptera | Anthomyzidae | x |  |  |  |  |  |  |
| Diptera | Asilidae |  |  | x |  |  |  |  |
| Diptera | Athericidae |  |  |  |  |  |  | x |
| Diptera | Bibionidae | x |  |  |  |  |  |  |
| Diptera | Bolitophilidae |  |  |  |  |  | x |  |
| Diptera | Bombyliidae | x |  |  |  |  |  |  |
| Diptera | Calliphoridae |  |  |  |  |  | x |  |
| Diptera | Carnidae |  |  |  | x |  |  |  |
| Diptera | Cecidomyiidae | x |  |  |  |  |  |  |
| Diptera | Ceratopogonidae | x |  |  |  |  |  |  |
| Diptera | Chamaemyiidae |  |  | x |  |  |  |  |
| Diptera | Chaoboridae |  |  |  |  |  |  | x |
| Diptera | Chironomidae | x |  |  |  |  |  |  |
| Diptera | Chloropidae | x |  |  |  |  |  |  |
| Diptera | Chyromyidae |  |  |  |  |  |  | x |
| Diptera | Clusiidae | x |  |  |  |  |  |  |
| Diptera | Conopidae | x |  |  |  |  |  |  |
| Diptera | Culicidae | x |  |  |  |  |  |  |
| Diptera | Cylindrotomidae | x |  |  |  |  |  |  |
| Diptera | Diadocidiidae |  |  |  |  |  | x |  |
| Diptera | Diastatidae |  |  |  |  |  |  | x |
| Diptera | Ditomyiidae |  |  |  |  |  |  | x |
| Diptera | Dixidae | x |  |  |  |  |  |  |
| Diptera | Dolichopodidae |  |  | x |  |  |  |  |
| Diptera | Drosophilidae | x |  |  |  |  |  |  |
| Diptera | Dryomyzidae |  |  |  |  |  | x |  |
| Diptera | Empididae |  |  | x |  |  |  |  |
| Diptera | Ephydridae | x |  |  |  |  |  |  |
| Diptera | Fanniidae |  |  |  |  |  | x |  |
| Diptera | Heleomyzidae |  |  |  |  |  | x |  |
| Diptera | Hippoboscidae |  |  |  | x |  |  |  |
| Diptera | Hybotidae |  |  | x |  |  |  |  |
| Diptera | Iteaphila |  |  |  |  |  |  | x |
| Diptera | Keroplatidae |  |  |  |  |  | x |  |
| Diptera | Lauxaniidae |  |  |  |  |  | x |  |
| Diptera | Limoniidae | x |  |  |  |  |  |  |
| Diptera | Lonchaeidae | x |  |  |  |  |  |  |
| Diptera | Lonchopteridae |  |  |  |  |  | x |  |
| Diptera | Megamerinidae |  |  | x |  |  |  |  |
| Diptera | Micropezidae |  |  | x |  |  |  |  |
| Diptera | Milichiidae |  |  |  | x |  |  |  |
| Diptera | Muscidae |  |  |  |  |  | x |  |
| Diptera | Mycetophilidae |  |  |  |  |  | x |  |
| Diptera | Opomyzidae | x |  |  |  |  |  |  |
| Diptera | Pallopteridae | x |  |  |  |  |  |  |
| Diptera | Pediciidae |  |  |  |  |  |  | x |
| Diptera | Phoridae |  |  |  |  |  | x |  |
| Diptera | Piophilidae |  |  |  |  |  | x |  |
| Diptera | Pipunculidae |  |  |  |  | x |  |  |
| Diptera | Platypezidae |  |  |  |  |  | x |  |
| Diptera | Platystomatidae |  |  |  |  |  |  | x |
| Diptera | Polleniidae |  |  |  |  |  |  | x |
| Diptera | Pseudopomyzidae |  |  |  |  |  |  | x |
| Diptera | Psilidae | x |  |  |  |  |  |  |
| Diptera | Psychodidae | x |  |  |  |  |  |  |
| Diptera | Ptychopteridae | x |  |  |  |  |  |  |
| Diptera | Rhagionidae |  |  | x |  |  |  |  |
| Diptera | Rhiniidae |  |  |  |  |  |  | x |
| Diptera | Rhinophoridae |  |  |  | x |  |  |  |
| Diptera | Sarcophagidae |  |  |  |  |  | x |  |
| Diptera | Scathophagidae |  |  | x |  |  |  |  |
| Diptera | Scatopsidae |  |  |  |  |  | x |  |
| Diptera | Sciaridae |  |  |  |  |  | x |  |
| Diptera | Sciomyzidae |  |  |  | x |  |  |  |
| Diptera | Sepsidae |  |  |  |  |  | x |  |
| Diptera | Simuliidae |  |  |  | x |  |  |  |
| Diptera | Sphaeroceridae |  |  |  |  |  | x |  |
| Diptera | Stratiomyidae | x |  |  |  |  |  |  |
| Diptera | Syrphidae | x | x |  |  |  |  |  |
| Diptera | Tabanidae |  |  |  | x |  |  |  |
| Diptera | Tachinidae |  |  |  | x |  |  |  |
| Diptera | Tephritidae | x |  |  |  |  |  |  |
| Diptera | Thaumaleidae | x |  |  |  |  |  |  |
| Diptera | Therevidae |  |  | x |  |  |  |  |
| Diptera | Tipulidae | x |  |  |  |  |  |  |
| Diptera | Trichoceridae |  |  |  |  |  | x |  |
| Diptera | Ulidiidae |  |  |  |  |  | x |  |
| Entomobryomorpha |  |  |  |  |  |  | x |  |
| Entomobryomorpha | Entomobryidae |  |  |  |  |  | x |  |
| Entomobryomorpha | Isotomidae |  |  |  |  |  | x |  |
| Entomobryomorpha | Tomoceridae |  |  |  |  |  | x |  |
| Ephemeroptera |  |  |  |  |  |  | x |  |
| Ephemeroptera | Baetidae |  |  |  |  |  | x |  |
| Ephemeroptera | Ephemeridae |  |  |  |  |  | x |  |
| Ephemeroptera | Heptageniidae |  |  |  |  |  | x |  |
| Ephemeroptera | Leptophlebiidae |  |  |  |  |  | x |  |
| Hemiptera | Acanthosomatidae | x |  |  |  |  |  |  |
| Hemiptera | Adelgidae | x |  |  |  |  |  |  |
| Hemiptera | Aleyrodidae | x |  |  |  |  |  |  |
| Hemiptera | Alydidae | x |  |  |  |  |  |  |
| Hemiptera | Anthocoridae |  |  | x |  |  |  |  |
| Hemiptera | Aphididae | x |  |  |  |  |  |  |
| Hemiptera | Aphrophoridae | x |  |  |  |  |  |  |
| Hemiptera | Berytidae | x |  |  |  |  |  |  |
| Hemiptera | Cercopidae | x |  |  |  |  |  |  |
| Hemiptera | Cicadellidae | x |  |  |  |  |  |  |
| Hemiptera | Cixiidae | x |  |  |  |  |  |  |
| Hemiptera | Coreidae | x |  |  |  |  |  |  |
| Hemiptera | Cydnidae | x |  |  |  |  |  |  |
| Hemiptera | Cymidae | x |  |  |  |  |  |  |
| Hemiptera | Delphacidae | x |  |  |  |  |  |  |
| Hemiptera | Issidae | x | x |  |  |  |  |  |
| Hemiptera | Liviidae | x | x |  |  |  |  |  |
| Hemiptera | Lygaeidae | x | x |  |  |  |  |  |
| Hemiptera | Miridae | x |  |  |  |  |  |  |
| Hemiptera | Nabidae |  |  | x |  |  |  |  |
| Hemiptera | Pentatomidae | x |  |  |  |  |  |  |
| Hemiptera | Psyllidae | x |  |  |  |  |  |  |
| Hemiptera | Reduviidae |  |  | x |  |  |  |  |
| Hemiptera | Rhopalidae | x |  |  |  |  |  |  |
| Hemiptera | Scutelleridae | x |  |  |  |  |  |  |
| Hemiptera | Stenocephalidae | x |  |  |  |  |  |  |
| Hemiptera | Tingidae | x |  |  |  |  |  |  |
| Hemiptera | Triozidae | x |  |  |  |  |  |  |
| Hymenoptera |  |  |  |  |  |  |  | x |
| Hymenoptera | Ampulicidae |  |  | x |  |  |  |  |
| Hymenoptera | Andrenidae | x | x |  |  |  |  |  |
| Hymenoptera | Aphelinidae | x | x |  |  |  |  |  |
| Hymenoptera | Apidae | x | x |  |  |  |  |  |
| Hymenoptera | Argidae | x | x |  |  |  |  |  |
| Hymenoptera | Astatidae |  |  |  |  |  |  | x |
| Hymenoptera | Bembicidae |  |  |  |  |  |  | x |
| Hymenoptera | Bethylidae |  |  |  |  | x |  |  |
| Hymenoptera | Braconidae |  |  |  |  | x |  |  |
| Hymenoptera | Cephidae | x | x |  |  |  |  |  |
| Hymenoptera | Ceraphronidae |  |  |  |  | x |  |  |
| Hymenoptera | Chalcididae |  |  |  |  | x |  |  |
| Hymenoptera | Chrysididae |  |  |  |  | x |  |  |
| Hymenoptera | Cimbicidae | x |  |  |  |  |  |  |
| Hymenoptera | Colletidae | x | x |  |  |  |  |  |
| Hymenoptera | Crabronidae |  |  | x |  |  |  |  |
| Hymenoptera | Cynipidae | x | x |  |  |  |  |  |
| Hymenoptera | Diapriidae |  |  |  |  | x |  |  |
| Hymenoptera | Diprionidae | x | x |  |  |  |  |  |
| Hymenoptera | Dryinidae |  |  |  |  | x |  |  |
| Hymenoptera | Encyrtidae |  |  |  |  | x |  |  |
| Hymenoptera | Eulophidae |  |  |  |  | x |  |  |
| Hymenoptera | Eupelmidae |  |  |  |  | x |  |  |
| Hymenoptera | Eurytomidae |  |  |  |  | x |  |  |
| Hymenoptera | Evaniidae |  |  |  |  | x |  |  |
| Hymenoptera | Figitidae |  |  |  |  | x |  |  |
| Hymenoptera | Formicidae |  |  | x |  |  |  |  |
| Hymenoptera | Gasteruptiidae |  |  |  |  | x |  |  |
| Hymenoptera | Halictidae | x | x |  |  |  |  |  |
| Hymenoptera | Heptamelidae | x | x |  |  |  |  |  |
| Hymenoptera | Ichneumonidae |  |  |  |  | x |  |  |
| Hymenoptera | Megachilidae | x | x |  |  |  |  |  |
| Hymenoptera | Megaspilidae |  |  |  |  | x |  |  |
| Hymenoptera | Melittidae | x | x |  |  |  |  |  |
| Hymenoptera | Mellinidae | x | x |  |  |  |  |  |
| Hymenoptera | Mymaridae |  |  |  |  | x |  |  |
| Hymenoptera | Ormyridae |  |  |  |  | x |  |  |
| Hymenoptera | Pamphiliidae | x | x |  |  |  |  |  |
| Hymenoptera | Pemphredonidae |  |  |  |  | x |  |  |
| Hymenoptera | Perilampidae |  |  |  |  |  |  | x |
| Hymenoptera | Philanthidae |  |  | x |  |  |  |  |
| Hymenoptera | Platygastridae |  |  |  |  | x |  |  |
| Hymenoptera | Pompilidae |  |  |  |  | x |  |  |
| Hymenoptera | Proctotrupidae |  |  |  |  | x |  |  |
| Hymenoptera | Psenidae |  |  | x |  |  |  |  |
| Hymenoptera | Pteromalidae |  |  |  |  | x |  |  |
| Hymenoptera | Siricidae |  |  |  |  |  |  | x |
| Hymenoptera | Sphecidae |  |  |  |  | x |  |  |
| Hymenoptera | Tenthredinidae | x | x |  |  |  |  |  |
| Hymenoptera | Torymidae | x | x |  |  |  |  |  |
| Hymenoptera | Trichogrammatidae |  |  |  |  | x |  |  |
| Hymenoptera | Vespidae |  |  | x |  |  |  |  |
| Hymenoptera | Xyelidae |  |  |  |  |  |  | x |
| Isopoda | Armadillidiidae |  |  |  |  |  | x |  |
| Isopoda | Philosciidae |  |  |  |  |  | x |  |
| Ixodida | Ixodidae |  |  | x |  |  |  |  |
| Julida | Julidae |  |  |  |  |  | x |  |
| Lepidoptera |  |  |  |  |  |  |  | x |
| Lepidoptera | Adelidae | x | x |  |  |  |  |  |
| Lepidoptera | Alucitidae | x | x |  |  |  |  |  |
| Lepidoptera | Argyresthiidae | x | x |  |  |  |  |  |
| Lepidoptera | Autostichidae | x | x |  |  |  |  |  |
| Lepidoptera | Batrachedridae | x | x |  |  |  |  |  |
| Lepidoptera | Bedelliidae | x | x |  |  |  |  |  |
| Lepidoptera | Blastobasidae | x | x |  |  |  |  |  |
| Lepidoptera | Bucculatricidae | x | x |  |  |  |  |  |
| Lepidoptera | Choreutidae | x | x |  |  |  |  |  |
| Lepidoptera | Coleophoridae | x | x |  |  |  |  |  |
| Lepidoptera | Cosmopterigidae | x | x |  |  |  |  |  |
| Lepidoptera | Cossidae | x | x |  |  |  |  |  |
| Lepidoptera | Crambidae | x | x |  |  |  |  |  |
| Lepidoptera | Depressariidae | x | x |  |  |  |  |  |
| Lepidoptera | Douglasiidae | x | x |  |  |  |  |  |
| Lepidoptera | Drepanidae | x | x |  |  |  |  |  |
| Lepidoptera | Elachistidae | x | x |  |  |  |  |  |
| Lepidoptera | Epermeniidae | x | x |  |  |  |  |  |
| Lepidoptera | Erebidae | x | x |  |  |  |  |  |
| Lepidoptera | Gelechiidae | x | x |  |  |  |  |  |
| Lepidoptera | Geometridae | x | x |  |  |  |  |  |
| Lepidoptera | Glyphipterigidae | x | x |  |  |  |  |  |
| Lepidoptera | Gracillariidae | x | x |  |  |  |  |  |
| Lepidoptera | Heliozelidae | x | x |  |  |  |  |  |
| Lepidoptera | Hepialidae | x | x |  |  |  |  |  |
| Lepidoptera | Hesperiidae | x | x |  |  |  |  |  |
| Lepidoptera | Incurvariidae | x | x |  |  |  |  |  |
| Lepidoptera | Lasiocampidae | x | x |  |  |  |  |  |
| Lepidoptera | Lycaenidae | x | x |  |  |  |  |  |
| Lepidoptera | Lyonetiidae | x | x |  |  |  |  |  |
| Lepidoptera | Lypusidae | x | x |  |  |  |  |  |
| Lepidoptera | Micropterigidae | x | x |  |  |  |  |  |
| Lepidoptera | Momphidae | x | x |  |  |  |  |  |
| Lepidoptera | Nepticulidae | x | x |  |  |  |  |  |
| Lepidoptera | Noctuidae | x | x |  |  |  |  |  |
| Lepidoptera | Nolidae | x | x |  |  |  |  |  |
| Lepidoptera | Notodontidae | x | x |  |  |  |  |  |
| Lepidoptera | Nymphalidae | x | x |  |  |  |  |  |
| Lepidoptera | Oecophoridae | x | x |  |  |  |  |  |
| Lepidoptera | Opostegidae | x | x |  |  |  |  |  |
| Lepidoptera | Papilionidae | x | x |  |  |  |  |  |
| Lepidoptera | Pieridae | x | x |  |  |  |  |  |
| Lepidoptera | Plutellidae | x | x |  |  |  |  |  |
| Lepidoptera | Praydidae | x | x |  |  |  |  |  |
| Lepidoptera | Prodoxidae | x | x |  |  |  |  |  |
| Lepidoptera | Psychidae | x | x |  |  |  |  |  |
| Lepidoptera | Pterophoridae | x | x |  |  |  |  |  |
| Lepidoptera | Pyralidae | x | x |  |  |  |  |  |
| Lepidoptera | Roeslerstammiidae | x | x |  |  |  |  |  |
| Lepidoptera | Schreckensteiniidae | x | x |  |  |  |  |  |
| Lepidoptera | Scythrididae | x | x |  |  |  |  |  |
| Lepidoptera | Scythropiidae | x | x |  |  |  |  |  |
| Lepidoptera | Sesiidae | x | x |  |  |  |  |  |
| Lepidoptera | Sphingidae | x | x |  |  |  |  |  |
| Lepidoptera | Stathmopodidae | x | x |  |  |  |  |  |
| Lepidoptera | Tineidae | x | x |  |  |  |  |  |
| Lepidoptera | Tischeriidae | x | x |  |  |  |  |  |
| Lepidoptera | Tortricidae | x | x |  |  |  |  |  |
| Lepidoptera | Yponomeutidae | x | x |  |  |  |  |  |
| Lepidoptera | Ypsolophidae | x | x |  |  |  |  |  |
| Lepidoptera | Zygaenidae | x | x |  |  |  |  |  |
| Lithobiomorpha |  |  |  | x |  |  |  |  |
| Mecoptera | Panorpidae | x |  |  |  |  |  |  |
| Mesostigmata |  |  |  | x |  |  |  |  |
| Mesostigmata | Ascidae |  |  | x |  |  |  |  |
| Mesostigmata | Digamasellidae |  |  | x |  |  |  |  |
| Mesostigmata | Macrochelidae |  |  | x |  |  |  |  |
| Mesostigmata | Parasitidae |  |  | x |  |  |  |  |
| Neuroptera | Chrysopidae |  |  | x |  |  |  |  |
| Neuroptera | Coniopterygidae |  |  | x |  |  |  |  |
| Neuroptera | Hemerobiidae |  |  | x |  |  |  |  |
| Neuroptera | Myrmeleontidae |  |  | x |  |  |  |  |
| Neuroptera | Osmylidae |  |  | x |  |  |  |  |
| Neuroptera | Sisyridae |  |  | x |  |  |  |  |
| Odonata | Aeshnidae |  |  | x |  |  |  |  |
| Odonata | Calopterygidae |  |  | x |  |  |  |  |
| Odonata | Coenagrionidae |  |  | x |  |  |  |  |
| Odonata | Gomphidae |  |  | x |  |  |  |  |
| Odonata | Platycnemididae |  |  | x |  |  |  |  |
| Opiliones |  |  |  | x |  |  |  |  |
| Opiliones | Phalangiidae |  |  | x |  |  |  |  |
| Opiliones | Sclerosomatidae |  |  | x |  |  |  |  |
| Orthoptera | Acrididae | x |  |  |  |  |  |  |
| Orthoptera | Gryllidae | x |  |  |  |  |  |  |
| Orthoptera | Tetrigidae | x |  |  |  |  |  |  |
| Orthoptera | Tettigoniidae | x |  |  |  |  |  |  |
| Plecoptera | Chloroperlidae | x |  |  |  |  |  |  |
| Plecoptera | Leuctridae |  |  |  |  |  | x |  |
| Plecoptera | Nemouridae |  |  |  |  |  | x |  |
| Plecoptera | Perlodidae |  |  |  |  |  | x |  |
| Plecoptera | Taeniopterygidae |  |  |  |  |  | x |  |
| Poduromorpha |  |  |  |  |  |  | x |  |
| Psocodea | Caeciliusidae |  |  |  |  |  | x |  |
| Psocodea | Paracaeciliidae |  |  |  |  |  | x |  |
| Psocodea | Stenopsocidae |  |  |  |  |  | x |  |
| Raphidioptera | Raphidiidae |  |  | x |  |  |  |  |
| Symphypleona | Bourletiellidae |  |  |  |  |  | x |  |
| Symphypleona | Dicyrtomidae |  |  |  |  |  | x |  |
| Symphypleona | Katiannidae |  |  |  |  |  | x |  |
| Symphypleona | Sminthuridae |  |  |  |  |  | x |  |
| Thysanoptera | Thripidae | x |  |  |  |  |  |  |
| Trichoptera | Beraeidae |  |  |  |  |  | x |  |
| Trichoptera | Glossosomatidae |  |  |  |  |  | x |  |
| Trichoptera | Hydropsychidae |  |  |  |  |  | x |  |
| Trichoptera | Lepidostomatidae |  |  |  |  |  | x |  |
| Trichoptera | Leptoceridae |  |  |  |  |  | x |  |
| Trichoptera | Limnephilidae |  |  |  |  |  | x |  |
| Trichoptera | Philopotamidae |  |  |  |  |  | x |  |
| Trichoptera | Phryganeidae |  |  |  |  |  | x |  |
| Trichoptera | Polycentropodidae |  |  |  |  |  | x |  |
| Trichoptera | Sericostomatidae |  |  |  |  |  | x |  |
| Trombidiformes |  |  |  | x |  |  |  |  |
| Trombidiformes | Anystidae |  |  | x |  |  |  |  |
| Trombidiformes | Microtrombidiidae |  |  | x |  |  |  |  |

Table S2 BIN numbers for each combination of functional and taxonomic groups and relative proportions of BIN numbers for each group.

|  | Cole-optera | Lepid-optera | Hyme-noptera | Diptera | Other Orders | Total within functional group | Percentage of total BIN number (%) | |
| --- | --- | --- | --- | --- | --- | --- | --- | --- |
| Herbivores | 359 | 1138 | 429 | 1238 | 412 | 3576 | 49 | |
| Pollinators^1^ | 16 | 1138 | 428 | 87 | 9 | 1678 | 23 | |
| Predators | 336 | 0 | 172 | 185 | 193 | 886 | 12 | |
| Parasitoids^2^ | 0 | 0 | 1053 | 227 | 0 | 1280 | 18 | |
| Detritivores | 120 | 0 | 0 | 773 | 147 | 1040 | 14 | |
| None | 209 | 28 | 108 | 174 | 0 | 519 | 7 | |
| Total within tax. group | 1024 | 1166 | 1762 | 2597 | 752 | 7301 | 100 | |
| Percentage of total BIN number (%) | 14 | 16 | 24 | 36 | 23 | 100 |  | |
| ^1^Pollinators are not included in calculation of total BIN numbers within taxonomic groups, since all pollinators also belong to the group of herbivores. For this reason, the sum of percentages in the right column exceeds 100.  ^2^We summarized parasitoids and parasites to one group, containing 1090 parasitoids and 190 parasites. | | | | | | | |  |

Table S3 Numbers and relative proportions of plant species with Ellenberg indicator values.

| Ellenberg indicator values | Number of species | Percentage of total (%) |
| --- | --- | --- |
| Light | 707 | 71 |
| Temperature | 533 | 54 |
| Continentality | 641 | 65 |
| Moisture | 656 | 66 |
| Soil pH | 548 | 55 |
| Nutrients | 656 | 66 |

Table S4 Summary statistics of plant and arthropod numbers and Ellenberg indicator values for the 179 sampling sites.

|  | Mean | Standard deviation | Minimum value | Maximum value |
| --- | --- | --- | --- | --- |
| Number of arthropod BINs | 824.09 | 176.53 | 438 | 1246 |
| Number of plant species | 114.20 | 29.33 | 52 | 204 |
|  |  |  |  |  |
| Ellenberg indicator values: |  |  |  |  |
| Light | 6.45 | 0.39 | 5.52 | 7.29 |
| Temperature | 5.51 | 0.22 | 4.78 | 5.96 |
| Continentality | 3.67 | 0.16 | 3.30 | 4.33 |
| Moisture | 5.22 | 0.36 | 4.38 | 6.24 |
| Soil pH | 6.33 | 0.59 | 4.34 | 7.27 |
| Nutrients | 5.59 | 0.39 | 4.25 | 6.60 |

Table S5 Distribution of species numbers across plant families (absolute numbers and relative proportions) calculated for all species recorded (left) and for host plants for monophagous herbivores (right). Families containing ≤10 species in the full dataset were assigned to ‘Other’.

|  | All plant species | | Host plant species of monophagous herbivores | |
| --- | --- | --- | --- | --- |
| Family | Species per family | Species (%) | Species per family | Species (%) |
| Asteraceae | 111 | 11.2 | 33 | 6.7 |
| Poaceae | 88 | 8.9 | 46 | 9.4 |
| Rosaceae | 60 | 6.1 | 34 | 6.9 |
| Fabaceae | 57 | 5.8 | 43 | 8.8 |
| Lamiaceae | 47 | 4.8 | 21 | 4.3 |
| Brassicaceae | 39 | 4.0 | 10 | 2.0 |
| Caryophyllaceae | 37 | 3.7 | 11 | 2.2 |
| Ranunculaceae | 29 | 2.9 | 14 | 2.9 |
| Scrophulariaceae | 28 | 2.8 | 15 | 3.1 |
| Apiaceae | 26 | 2.6 | 2 | 0.4 |
| Cyperaceae | 26 | 2.6 | 24 | 4.9 |
| Polygonaceae | 19 | 1.9 | 18 | 3.7 |
| Caprifoliaceae | 18 | 1.8 | 14 | 2.9 |
| Boraginaceae | 16 | 1.6 | 5 | 1.0 |
| Geraniaceae | 15 | 1.5 | 13 | 2.6 |
| Pinaceae | 15 | 1.5 | 13 | 2.6 |
| Juncaceae | 14 | 1.4 | 14 | 2.9 |
| Rubiaceae | 14 | 1.4 | 12 | 2.4 |
| Campanulaceae | 13 | 1.3 | 8 | 1.6 |
| Onagraceae | 12 | 1.2 | 10 | 2.0 |
| Euphorbiaceae | 11 | 1.1 | 9 | 1.8 |
| Other | 292 | 29.6 | 122 | 24.8 |
| Total | 987 | 100.0 | 491 | 100.0 |

Table S6 Unique and shared variance fractions of plant species composition (PLANT), land cover composition (LAND), climate (CLIM) and space in five functional and four taxonomic arthropod groups, as obtained in variation partitioning based on distance-based redundancy analysis. Sørensen index was used to calculate arthropod between-site dissimilarities. Predictive dissimilarities were calculated using the Sørensen index for plant species composition and Euclidean distances for land cover, climate and space.

|  | Herbivores | Pollinators | Predators | Parasitoids | Detritivores | Lepidoptera | Coleoptera | Hymenoptera | Diptera |
| --- | --- | --- | --- | --- | --- | --- | --- | --- | --- |
| PLANT | 0.106 | 0.090 | 0.148 | 0.063 | 0.070 | 0.073 | 0.101 | 0.063 | 0.111 |
| LAND | 0.035 | 0.017 | 0.011 | 0.021 | 0.023 | 0.022 | 0.017 | 0.018 | 0.031 |
| CLIM | 0.010 | 0.017 | 0.000 | 0.006 | 0.007 | 0.020 | 0.016 | 0.005 | 0.005 |
| SPACE | 0.013 | 0.010 | 0.000 | 0.021 | 0.012 | 0.013 | 0.006 | 0.015 | 0.014 |
| PLANT + LAND | 0.130 | 0.088 | 0.109 | 0.077 | 0.085 | 0.085 | 0.096 | 0.073 | 0.126 |
| LAND + CLIM | -0.003 | -0.002 | 0.000 | -0.002 | -0.002 | -0.003 | -0.002 | -0.001 | -0.002 |
| PLANT + CLIM | 0.035 | 0.038 | 0.012 | 0.021 | 0.024 | 0.036 | 0.040 | 0.019 | 0.029 |
| PLANT + SPACE | -0.003 | -0.003 | 0.000 | -0.003 | -0.003 | -0.003 | -0.002 | -0.003 | -0.004 |
| LAND + SPACE | 0.001 | 0.000 | 0.000 | 0.001 | 0.000 | 0.000 | 0.000 | 0.000 | 0.001 |
| CLIM + SPACE | 0.028 | 0.032 | 0.000 | 0.029 | 0.023 | 0.039 | 0.024 | 0.022 | 0.022 |
| PLANT + LAND + SPACE | -0.002 | -0.001 | 0.000 | -0.002 | -0.001 | -0.001 | -0.001 | -0.001 | -0.002 |
| PLANT + LAND + CLIM | 0.021 | 0.018 | 0.008 | 0.012 | 0.014 | 0.019 | 0.019 | 0.011 | 0.017 |
| LAND + CLIM + SPACE | -0.003 | -0.002 | 0.000 | -0.003 | -0.002 | -0.003 | -0.002 | -0.002 | -0.003 |
| PLANT + CLIM + SPACE | 0.032 | 0.030 | 0.007 | 0.027 | 0.024 | 0.030 | 0.027 | 0.023 | 0.030 |
| PLANT + LAND + CLIM + SPACE | 0.016 | 0.012 | 0.003 | 0.013 | 0.012 | 0.014 | 0.011 | 0.011 | 0.015 |
| Total adjusted R^2^ | 0.415 | 0.344 | 0.298 | 0.281 | 0.287 | 0.341 | 0.350 | 0.253 | 0.390 |

Table S7 Unique and shared variance fractions of Ellenberg indicator values (INDVAL) derived from plant species composition, land cover composition (LAND), climate (CLIM) and space in five functional and four taxonomic arthropod groups, as obtained in variation partitioning based on distance-based redundancy analysis. Sørensen index was used to calculate arthropod between-site dissimilarities. Predictive dissimilarities were calculated using the Sørensen index for plant species composition and Euclidean distances for land cover, climate and space. This version only uses Ellenberg indicator values and no information on plant species identity as predictor.

|  | Herbivores | Pollinators | Predators | Parasitoids | Detritivores | Lepidoptera | Coleoptera | Hymenoptera | Diptera |
| --- | --- | --- | --- | --- | --- | --- | --- | --- | --- |
| INDVAL | 0.091 | 0.078 | 0.115 | 0.048 | 0.078 | 0.060 | 0.083 | 0.048 | 0.101 |
| LAND | 0.082 | 0.047 | 0.048 | 0.051 | 0.049 | 0.053 | 0.050 | 0.046 | 0.074 |
| CLIM | 0.011 | 0.018 | 0.000 | 0.007 | 0.006 | 0.021 | 0.017 | 0.006 | 0.005 |
| SPACE | 0.013 | 0.009 | 0.000 | 0.020 | 0.012 | 0.012 | 0.005 | 0.014 | 0.013 |
| INDVAL + LAND | 0.083 | 0.058 | 0.073 | 0.047 | 0.060 | 0.054 | 0.063 | 0.045 | 0.083 |
| LAND + CLIM | -0.001 | -0.001 | 0.000 | -0.001 | -0.001 | -0.001 | -0.001 | -0.001 | -0.001 |
| INDVAL + CLIM | 0.034 | 0.038 | 0.012 | 0.019 | 0.025 | 0.035 | 0.039 | 0.018 | 0.029 |
| INDVAL + SPACE | -0.003 | -0.002 | 0.000 | -0.003 | -0.002 | -0.002 | -0.002 | -0.002 | -0.003 |
| LAND + SPACE | 0.000 | 0.000 | 0.000 | 0.000 | 0.000 | 0.000 | 0.000 | 0.000 | 0.000 |
| CLIM + SPACE | 0.028 | 0.032 | 0.000 | 0.030 | 0.021 | 0.039 | 0.024 | 0.023 | 0.021 |
| INDVAL + LAND + SPACE | -0.001 | -0.001 | 0.000 | -0.001 | -0.001 | -0.001 | -0.001 | -0.001 | -0.001 |
| INDVAL + LAND + CLIM | 0.019 | 0.017 | 0.008 | 0.011 | 0.013 | 0.017 | 0.018 | 0.011 | 0.016 |
| LAND + CLIM + SPACE | -0.001 | -0.001 | 0.000 | -0.001 | -0.001 | -0.001 | -0.001 | -0.001 | -0.001 |
| INDVAL + CLIM + SPACE | 0.032 | 0.030 | 0.006 | 0.026 | 0.026 | 0.030 | 0.026 | 0.022 | 0.031 |
| INDVAL + LAND + CLIM + SPACE | 0.014 | 0.011 | 0.003 | 0.011 | 0.010 | 0.012 | 0.010 | 0.010 | 0.013 |
| Total adjusted R^2^ | 0.399 | 0.331 | 0.265 | 0.266 | 0.295 | 0.329 | 0.332 | 0.238 | 0.380 |
